# Diagnostic Complexity of Adult‐Onset Still’s Disease in the Setting of Nosocomial Infection and Antibiotic Hypersensitivity

**DOI:** 10.1155/crii/4891053

**Published:** 2026-10-17

**Authors:** Mei-Chun Lee, Chao-Hsien Lee, Tzu-Han Weng, Ya-Hui Chang

**Affiliations:** ^1^ Department of Pharmacy MacKay Memorial Hospital Taipei Taiwan mmh.org.tw; ^2^ Critical Care Division Internal Medicine Department MacKay Memorial Hospital Taipei Taiwan mmh.org.tw; ^3^ Department of Dermatology MacKay Memorial Hospital Taipei Taiwan mmh.org.tw; ^4^ School of Medicine College of Medicine MacKay Medical University New Taipei City Taiwan mmu.edu.tw

## Abstract

Adult‐onset Still’s disease (AOSD) is a diagnosis of exclusion and may be difficult to recognize when infection and drug hypersensitivity coexist. A 31‐year‐old woman presented with high‐spiking fever, arthralgia, and a generalized eruption after ampicillin exposure. Initial investigations for infection and autoimmune disease were unrevealing. Her clinical course was subsequently complicated by severe nosocomial infection and recurrent eruptions during antibiotic treatment. Recurrent high‐spiking fever and a fever‐associated eruption after apparent clinical improvement of infection supported AOSD as the working diagnosis. However, uncertainty regarding the rash and overlapping infectious and drug‐related features limited confident retrospective confirmation of the Yamaguchi classification criteria. Following infection control, intravenous methylprednisolone pulse therapy followed by cyclosporine resulted in rapid clinical improvement. This case illustrates the diagnostic complexity of AOSD in the setting of nosocomial infection and antibiotic hypersensitivity.

## 1. Clinical Case

A 31‐year‐old woman underwent dental root canal treatment and was prescribed ampicillin. The following day, she developed a generalized, nonpruritic eruption. On day 3, fever, progression of the eruption, and bilateral knee arthralgia prompted hospital admission, where empirical treatment with moxifloxacin was initiated.

During the early hospital course, the eruption partially subsided, but high‐spiking fever persisted, reaching 40°C. On hospital day 6, erythematous macules and patches recurred on the face, trunk, and extremities (Figure 1a). Abdominal ultrasonography showed borderline splenomegaly and a small left pleural effusion. The serum ferritin level was mildly elevated to 94.14 ng/mL. Neutrophilic leucocytosis was present, with a leucocyte count of 11.1 × 10^3^/µL and 88% neutrophils. Viral studies for HSV, HAV, HBV, HCV, HIV, and CMV were negative, while EBV serology was consistent with past infection (VCA IgG >750 U/mL, VCA IgM 10.7 U/mL, EA IgG <5 U/mL, and EBNA IgG 526 U/mL). Autoimmune investigations, including ANA, anti‐dsDNA, ENA, ANCA, and rheumatoid factor, were negative. Ga‐67 inflammatory scintigraphy revealed no significant abnormal uptake, suggestive of an occult inflammatory or malignant process. These findings reduced the likelihood of several competing diagnoses. With these alternative diagnoses considered less likely, adult‐onset Still’s disease (AOSD) was suspected based on the overall clinical presentation and the Yamaguchi criteria, prompting the initiation of systemic corticosteroid therapy. Oral prednisolone (5 mg twice daily) was administered for 1 day, followed by intravenous hydrocortisone (100 mg twice daily) for 1 day, and subsequently switched to intravenous methylprednisolone (40 mg twice daily). The clinical timeline was shown in Figure 2.

**Figure 1:**
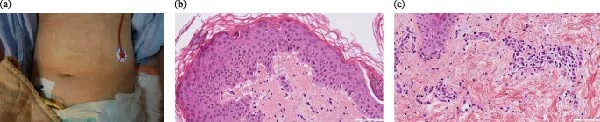
Clinical and histopathological findings of the cutaneous eruption. (a) Confluent erythematous macules and patches over the trunk during a recurrent pruritic eruption. (b) Clustered dyskeratotic keratinocytes in the upper epidermis. (c) Superficial dermal inflammatory infiltrates with karyorrhectic debris consistent with fragmented neutrophils. Hematoxylin and eosin staining.

**Figure 2:**
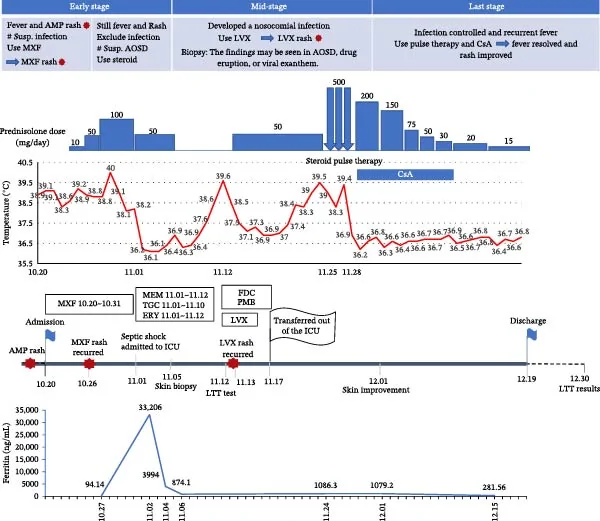
Clinical course after hospital admission. The timeline shows the temporal relationships among body temperature, serum ferritin levels, episodes of cutaneous eruption, antimicrobial exposure, and immunosuppressive treatment. AMP, ampicillin; AOSD, adult‐onset Still’s disease; CsA, cyclosporine; ERY, erythromycin; FDC, cefiderocol; LTT, lymphocyte transformation test; LVX, levofloxacin; MEM, meropenem; MXF, moxifloxacin; PMB, polymyxin B; TGC, tigecycline.

On hospital day 12, the patient developed septic shock and hypoxemic respiratory failure, requiring admission to the intensive care unit and mechanical ventilation. Intravenous hydrocortisone (50 mg every 6 h) was administered for 5 days in accordance with sepsis management guidelines. Chest radiography demonstrated rapidly progressive bilateral infiltrates. Urinary antigen tests were positive for *Streptococcus pneumoniae* and *Legionella pneumophila*. Procalcitonin subsequently increased to 15.24 ng/mL. Chest CT demonstrated large bilateral lower‐lung consolidations with surrounding ground‐glass opacities and large bilateral pleural effusions, supporting the diagnosis of severe pneumonia. The pneumonia was considered hospital‐acquired because it developed on hospital day 12, more than 48 h after admission, with no clinical evidence of pneumonia at presentation. Antimicrobial treatment was broadened to meropenem, tigecycline, and erythromycin. Concurrently, ferritin increased to 33,206 ng/mL, accompanied by a hemoglobin level of 9.5 g/dL, a white blood cell count of 19.9 × 10^3^/μL, thrombocytopenia (platelet count, 121 × 10^3^/μL), mild hypofibrinogenemia (161.1 mg/dL), elevated LDH (up to 3755 U/L), and marked liver dysfunction (AST/ALT, 727/566 U/L).

A skin biopsy performed on hospital day 16 showed dyskeratotic keratinocytes in the upper epidermis and a superficial dermal inflammatory infiltrate with karyorrhectic debris (Figure 1b,c). These findings were compatible with AOSD‐associated cutaneous disease but were not specific; a drug eruption or viral exanthem could not be excluded. Following the antimicrobial treatment, the patient improved and became afebrile.

On hospital day 23, the patient developed recurrent fever, and a blood sample was collected for an LTT and sent to an external reference laboratory to assess whether the eruption was drug‐related. Concurrently, blood cultures yielded the carbapenem‐resistant *Acinetobacter baumannii* complex. The carbapenem‐resistant *Acinetobacter baumannii* complex bacteremia was suspected to be catheter‐related. Antimicrobial therapy was changed to cefiderocol, polymyxin B, and levofloxacin. The morning after levofloxacin initiation, confluent, pruritic, and erythematous macules and patches developed over the trunk and extremities. Dermatological assessment favored AOSD‐associated cutaneous disease, although a maculopapular drug eruption could not be excluded. Levofloxacin was therefore discontinued, while methylprednisolone (40 mg once daily) was restarted. Following catheter replacement and modification of antimicrobial therapy, the patient’s clinical condition gradually improved. She was successfully extubated on hospital day 25, and blood cultures became negative with resolution of the fever on day 26. She was subsequently transferred out of the intensive care unit on day 30.

At the time of transfer out of the ICU, procalcitonin had decreased to 1.37 ng/mL, and the white blood cell count had declined from 19.9 × 10^3^/μL at ICU admission to 9.9 × 10^3^/μL. Follow‐up chest radiography on hospital day 33 showed only mild coarsening of the bilateral lung markings. The patient remained hemodynamically stable (blood pressure, 112/62 mmHg) and maintained an SpO_2_ of 96%–98% on room air.

However, high‐spiking fever recurred on hospital day 34, accompanied by a rash that became more prominent during the febrile episodes. A 3‐day course of intravenous methylprednisolone pulse therapy (500 mg once daily) was administered from hospital days 38–40, followed by cyclosporine (50 mg every 12 h) (Figure 2). The fever rapidly resolved, and the eruption markedly improved following corticosteroid pulse therapy.

The LTT results became available after the patient was discharged. The assay assessed drug‐induced lymphocyte activation based on granulysin (GNLY) and granzyme B (GZMB) responses. Positive responses were observed for ampicillin (GNLY, 1.63‐fold and GZMB, 2.31‐fold) and moxifloxacin (GNLY, 2.91‐fold and GZMB, 3.88‐fold), based on laboratory reference cutoffs of <1.2‐fold for GNLY and ≤1.5‐fold for GZMB. A LTT obtained before levofloxacin exposure was positive for ampicillin and moxifloxacin, supporting delayed‐type hypersensitivity. Levofloxacin was not included in the test; therefore, the rash occurring after levofloxacin exposure could not be confirmed as a drug hypersensitivity reaction based on the LTT findings.

The patient remained clinically stable during outpatient follow‐up. Oral prednisolone 5 mg bid was continued for 2 months after discharge and then discontinued. No recurrence of the skin eruption was observed during subsequent follow‐up.

## 2. Discussion

As the predominant causes of fever and cutaneous manifestations appeared to evolve throughout the clinical course, the following discussion is organized according to the clinical timeline, as shown in Figure 2.

### 2.1. Early Stage: Exclusion of Alternative Diagnoses and Consideration of AOSD

During the early stage of hospitalization, AOSD was considered based on the overall clinical presentation, including fever ≥39°C, neutrophilic leucocytosis, a fever‐associated eruption, splenomegaly, and negative rheumatoid factor and antinuclear antibody tests, which were considered to fulfill the Yamaguchi [1] classification criteria. However, attribution of the early cutaneous eruption solely to AOSD remained uncertain because it occurred in the setting of antibiotic exposure and may have been concomitantly influenced by drug hypersensitivity. Therefore, although the clinical presentation supported the consideration of AOSD, the cutaneous criterion should be interpreted with caution. AOSD is a diagnosis of exclusion, and the coexistence of infection and drug hypersensitivity further complicated the diagnostic process [2]. Accordingly, alternative infectious, autoimmune, and malignant causes were systematically evaluated during the early hospitalization period and were considered less likely based on the corresponding investigations.

### 2.2. Middle Stage: Severe Infection With Possible Concurrent AOSD Activity

MAS/secondary HLH was also considered during septic shock because of marked hyperferritinemia, liver dysfunction, elevated LDH, thrombocytopenia, and hypofibrinogenemia. However, these abnormalities occurred concurrently with severe infection and septic shock and could also have reflected infection‐associated systemic inflammation and coagulopathy. Therefore, MAS/secondary HLH could not be definitively confirmed or excluded.

The marked increase in ferritin accompanied by liver injury was not specific to AOSD and may have reflected both severe systemic inflammation and infection [3]. Infectious events have been proposed as potential triggers of AOSD onset or exacerbation, although a direct causal relationship has not been established [4–7]. Various viral and bacterial pathogens, including Epstein‐Barr virus, HIV, cytomegalovirus, parvovirus B19, adenovirus, influenza virus, SARS‐CoV‐2, *Mycoplasma pneumoniae*, and *Yersinia enterocolitica*, have been implicated, possibly through activation of the innate immune system [4–8]. Conversely, corticosteroids and biologic agents targeting IL‐1 or IL‐6, such as anakinra and tocilizumab, may increase the risk of serious infections. However, a causal relationship between corticosteroid therapy and subsequent nosocomial infections cannot be established based on this single case. Repeated reassessment after microbiological control was therefore essential.

### 2.3. Late Stage: Recurrent Inflammatory Activity and Subsequent Reassessment Based on LTT Results

Following transfer out of the ICU, the preceding infection appeared clinically controlled, with a substantial decrease in procalcitonin levels and improvement in pulmonary, respiratory, and hemodynamic status. Nevertheless, high‐spiking fever recurred, accompanied by a rash that became more prominent during the febrile episodes. This recurrence after apparent infection control raised concern for renewed inflammatory activity and prompted high‐dose methylprednisolone pulse therapy. The fever rapidly resolved, and the eruption markedly improved following treatment. This clinical course highlights the importance of reassessing recurrent inflammatory manifestations after infection control to distinguish infection from AOSD‐related inflammatory activity.

The cutaneous manifestations also raised the possibility of a concomitant antibiotic‐related maculopapular drug eruption. The LTT results, which became available after discharge, showed positive responses to ampicillin and moxifloxacin, providing supportive evidence of antibiotic hypersensitivity. In retrospect, the patient had developed suspected antibiotic‐related maculopapular rashes after 8 days of moxifloxacin treatment and after a single dose of levofloxacin. Such eruptions generally occur 4–21 days after drug initiation but may develop earlier following previous sensitization or exposure to a related drug [9, 10]. The more rapid onset of the rash following levofloxacin exposure raised the possibility that prior exposure to a drug of the same class (fluoroquinolones) may have contributed to its earlier appearance. However, levofloxacin was not included in the LTT; therefore, the association between levofloxacin exposure and the subsequent rash remained uncertain. Classic AOSD is associated with an evanescent, usually nonpruritic, salmon‐colored eruption. However, persistent pruritic eruptions with upper epidermis dyskeratosis are increasingly recognized as atypical manifestations of AOSD [11–13]. The differences between the skin manifestations of AOSD and maculopapular drug eruptions are summarized in Table 1. Although the LTT results were interpreted as supportive rather than definitive evidence of drug hypersensitivity, the clinical course, histopathological findings, antibiotic exposure, and LTT results suggested that AOSD‐associated cutaneous disease and antibiotic hypersensitivity may have coexisted.

**Table 1:** Comparison of cutaneous manifestations between AOSD and maculopapular drug eruptions.

Clinical features	AOSD rash [11–13]	Maculopapular drug eruption [9, 10] (exanthematous drug eruption)
Onset	Appears in ~80% of AOSD patients; 28% present with rash at disease onset	Typical 4–21 days after medication initiation; accelerated with prior sensitization
Course	Classic: appears with fever spikes, fades with defervescence. Atypical: persistent days to weeks, correlates with disease activity	Self‐limited; fades within days to 1 week after drug discontinuation
Fever type	Typical a high spiking quotidian fever (39°C), which occurs daily or occasionally peaks twice daily	Low‐grade fever^a^
Pruritus	Classic: nonpruritic. Atypical: persistent pruritic eruptions may occur.	Often mildly pruritic
Morphology	Typically: salmon‐colored and with macular or maculopapular lesions Atypical forms: persistent pruritic papules and plaques can occur, sometimes with linear or dermatomyositis‐like features.	Pink‐to‐red macules and papules that may coalesce into plaques, and may be accompanied
Distribution	Mainly on the trunk and proximal extremities, occasionally on the palms and soles	Widespread, symmetric; starts trunk/proximal extremities, may involve face
Histopathology	Interstitial and perivascular neutrophilic infiltrates, and in persistent lesions, dyskeratotic keratinocytes are found predominantly in the upper third of the epidermis.	A superficial perivascular lymphocytic infiltrate, sometimes with eosinophils, and dyskeratotic keratinocytes distributed throughout all layers of the epidermis

Abbreviation: AOSD, adult‐onset Still’s disease.

^a^Its severity varies depending on the clinical phenotype and severity of the drug reaction.

This case demonstrates the complex coexistence of AOSD‐related inflammatory activity, recurrent nosocomial infection, and antibiotic hypersensitivity, which may present with overlapping and clinically indistinguishable manifestations of fever and rash. Careful assessment of temporal relationships among fever, cutaneous manifestations, antimicrobial exposure, microbiological findings, and treatment response was essential for navigating this diagnostic complexity.

## Funding

No funding was received for this study.

## Conflicts of Interest

The authors declare no conflicts of interest.

## Data Availability

The data that support the findings of this study are available upon request from the corresponding author. The data are not publicly available due to privacy or ethical restrictions.
